# Computerized Symbol Digit Modalities Test in a Swiss Pediatric Cohort – Part 2: Clinical Implementation

**DOI:** 10.3389/fpsyg.2021.631535

**Published:** 2021-04-23

**Authors:** Marie-Noëlle Klein, Ursina Jufer-Riedi, Sarah Rieder, Céline Hochstrasser, Michelle Steiner, Li Mei Cao, Anthony Feinstein, Sandra Bigi, Karen Lidzba

**Affiliations:** ^1^Division of Child Neurology, Department of Pediatrics, University Children’s Hospital Bern, University of Bern, Bern, Switzerland; ^2^Department of Psychiatry, University of Toronto, Toronto, ON, Canada; ^3^Institute of Social and Preventive Medicine, University of Bern, Bern, Switzerland

**Keywords:** processing speed, cognitive function, pediatrics, computerized test, hospitalization

## Abstract

**Background:**

Information processing speed (IPS) is a marker for cognitive function. It is associated with neural maturation and increases during development. Traditionally, IPS is measured using paper and pencil tasks requiring fine motor skills. Such skills are often impaired in patients with neurological conditions. Therefore, an alternative that does not need motor dexterity is desirable. One option is the computerized symbol digit modalities test (c-SDMT), which requires the patient to verbally associate numbers with symbols.

**Methods:**

Eighty-six participants (8–16 years old; 45 male; 48 inpatients) were examined, 38 healthy and 48 hospitalized for a non-neurological disease. All participants performed the written SDMT, c-SDMT, and the Test of Non-verbal Intelligence Fourth Edition (TONI-4). Statistical analyses included a multivariate analysis of covariance (MANCOVA) for the effects of intelligence (IQ) and hospitalization on the performance of the SDMT and c-SDMT. A repeated measures analysis of variance (repeated measures ANOVA) was used to compare performance across c-SDMT trials between inpatients and outpatients.

**Results:**

The MANCOVA showed that hospitalization had a significant effect on IPS when measured with the SDMT (*p* = 0.04) but not with the c-SDMT (*p* = 0.68), while IQ (*p* = 0.92) had no effect on IPS. Age (*p* < 0.001) was the best predictor of performance of both tests. The repeated measures ANOVA revealed no significant difference in within-test performance (*p* = 0.06) between outpatient and inpatient participants in the c-SDMT.

**Conclusion:**

Performance of the c-SDMT is not confounded by hospitalization and gives within-test information. As a valid and reliable measure of IPS for children and adolescents, it is suitable for use in both inpatient and outpatient populations.

## Introduction

Information processing speed (IPS) is the brain’s potential to process information within a certain time period. One possible operationalization of IPS is the time it takes to react to a verbal, visual or auditory stimulus. This process includes the encoding, integration and retrieval of information, the decision to react, and finally, a response. Being a basic cognitive quality it can be used as a marker for cognitive dysfunction in patients with neurological disease (Fry and Hale, 2000). Neurological impairment in children and adolescents can have various causes, such as traumatic brain injury (TBI) (Babikian and Asarnow, 2009), epilepsy (Reilly et al., 2015), childhood stroke (Low et al., 2017), and multiple sclerosis (Bigi et al., 2017). A variety of tools have been developed for the assessment of IPS, ranging from simple to complex motor or verbal output (Holdnack et al., 2016).

One of the first standardized tests developed to assess cognitive function is the coding task, which was first documented in the early 1900s (Woodworth and Wells, 1911). Most versions are paper-and-pencil tests, where the participant matches as many digits with numbers as possible according to a given code within a given time. In the symbol digit modalities test (SDMT), a symbol–digit key has to be applied within 90 s to as many symbols as possible (Smith, 1973). The SDMT is easy to administer, but has several drawbacks. For example, the classical written version relies heavily on fine motor function. Thus, SDMT results of individuals with motor impairment (because of damage to the dominant arm, perfusion, etc.) have to be interpreted with caution. The standard version is prone to practice effects and therefore the results of repeated tests on the same participant are difficult to interpret (Smith, 1973). Over time, a range of alternative versions of the SDMT have been developed, among them an oral one (Smith, 1973), which has been applied in some studies on pediatric multiple sclerosis (Smerbeck et al., 2011; Hosseini et al., 2014; McKay et al., 2019; Margoni et al., 2020) and on pediatric traumatic brain injury (McCauley et al., 2014). The Brief Repeatable Neuropsychological Battery includes alternate versions of the SDMT (Rao, 1991), which are capable of reducing practice effects (Benedict et al., 2012). One of the newest versions is the tablet-based SDMT (T-SDMT). This test has been used in stroke patients, where it shows significant responsiveness and predictive value during rehabilitation (Tung et al., 2016).

In an attempt to further minimize the requirement for fine motor skills, the computerized symbol digit modalities test (c-SDMT) was developed and implemented in Canada (Akbar et al., 2011). It has also been standardized for use with children (Bigi et al., 2017). The c-SDMT requires a verbal rather than a motor response and is presented on a computer screen. In eight trials, each comprising nine symbol–digit pairings, the participant is asked to name the digit corresponding to each of the nine symbols shown on the screen as quickly as possible. The examiner documents response speed by a key-press or mouse-click at the end of every trial. The symbol–digit key can be held constant throughout the trials to assess the subject’s capacity to adapt to the task, or it can be changed both from trial to trial and from test to retest to minimize practice effects (Bigi et al., 2017). IPS can therefore be examined repeatedly during rehabilitation e.g., after traumatic brain injury. Thus, the c-SDMT can contribute to a tailored, individually focused rehabilitation strategy (Akbar et al., 2011; Bigi et al., 2017).

A study applying the c-SDMT in a cohort of adolescent multiple sclerosis patients revealed that their speed did not consistently increase from trial one to eight as it did in the control group (Bigi et al., 2017). This shows the advantage of the more detailed information the c-SDMT provides by being able to detect smaller alterations of IPS, not seen in the regular paper-and-pencil tests. Since IPS and cognitive function play a crucial role in school performance and activities of daily living (Weiss et al., 2016), detection and monitoring of these detailed aspects of IPS are valuable.

Based on increases in myelination (Bigi et al., 2017) and neural maturation, as well as short-term memory and reasoning abilities, IPS develops rapidly during childhood and adolescence as a function of age (Hale, 1990). In adulthood, performance in the SDMT stays relatively stable before it declines in parallel with a decrease in working memory (Kiely et al., 2014).

A rarely examined factor influencing IPS is hospitalization. Although both the SDMT and the c-SDMT are rapid and easy to administer bedside tools for the assessment of IPS, little is known about how hospitalization may influence performance of these tests. It is well recognized that hospitalization can cause a variety of reactions in children, which include distress, pain, the feeling of not being in control, being overwhelmed, discomfort (Perry, 2009) and fear (Kassam-Adams and Butler, 2017). Such reactions can occur regardless of the reason for hospitalization (Kassam-Adams and Butler, 2017). Furthermore, it is probable that even minor procedures can be frightening for children (Kassam-Adams and Butler, 2017). In particular, the time before and after surgery or other invasive interventions can be stressful and can cause anxiety as well as pain in children (Caldas et al., 2004).

Nervous or emotional conditions have been found to be significantly associated with lower SDMT scores, similarly to other non-specific health conditions (Kiely et al., 2014). Children’s coping strategies differ markedly between age groups. Older children have a better understanding of their disease and the necessity of the hospital stay and they can therefore adapt better to hospitalization (Hägglöf, 2007). The effects of hospitalization can be positively influenced and modified through aspects of care such as emotional support, child-adapted information, communication and environments (Linda Sari Siregar and Oktavinola Kaban, 2017) and by involving the children in various activities (Crnkoviæ et al., 2009), such as expressive art therapy (Siegel et al., 2016), clowns or games (Crnkoviæ et al., 2009). Hospitalization may impact a child’s emotional condition, concentration and cognitive ability in many ways and could thereby affect IPS (Scharfen et al., 2018) and consequently the child’s performance in the SDMT and c-SDMT.

Information processing speed is not only linked to everyday activities (Weiss et al., 2016), but is also known to correlate positively with general intelligence (Holdnack et al., 2016). The speed and efficiency of basic mental functions is highly correlated with the ability to perform complex cognitive tasks (Miller and Vernon, 1992; Hägglöf, 2007). IPS and working memory, as well as fluid reasoning, increase concomitantly during childhood and adolescence (Fry and Hale, 2000). It is known that 45% of age-related effects on fluid reasoning is mediated by differences in speed and working memory (Fry and Hale, 2000). On the neurological level, these associations can be explained by more efficient transmission of information from the frontal attention and working memory networks to temporal-parietal memory storage (Schubert et al., 2017). IPS is closely related to the capacity of working memory (Vernon, 1983), and there has been debate as to whether the two should be considered separate or dependent predictors of individual intelligence. Higher SDMT scores correlate positively with higher educational attainment (McCauley et al., 2014). In conclusion, individual differences in intelligence can partly be attributed to variances in IPS (Schubert et al., 2017). However, since in populations with neurological disease, compromised intelligence is more common than in healthy populations, it is desirable for an assessment of IPS to be as independent of general intelligence as possible.

The overall purpose of this study was to examine the clinical implementation of the c-SDMT in direct comparison to the original written SDMT.

Our main hypotheses were that IPS, measured by the SDMT or the c-SDMT, differs between neurologically healthy inpatient and outpatient children and adolescents (H1a). Furthermore, we predicted that the c-SDMT would be less prone to hospitalization effects than the SDMT (H1b). We also did not expect an effect of hospitalization on trial-to-trial behavior in the c-SDMT. Our second hypothesis was that IPS, when measured with the SDMT or the c-SDMT, is associated with intelligence (H2).

## Materials and Methods

### Study Design

The study conducted was a single-center pilot study with both cross-sectional and short-term longitudinal components. It took place at the University Children’s Hospital of the University of Bern in Switzerland. The study was approved by the ethics board of the canton of Bern (project ID 2018-00540) and was conducted in accordance with the ethical principles of the Declaration of Helsinki. Recruitment and testing of participants started in July 2018 and ended in December 2018. Data collection and analysis took place between January and June 2019.

### Participants

Eighty-six neurologically healthy pediatric participants were recruited in the region of Bern, Switzerland: 48 of them were inpatients of the surgical and medical ward at the University Children’s Hospital Bern, with a wide range of reasons for admission. The “outpatient” group had no obvious medical issues. Inclusion criteria: sufficient level of German, informed consent, age between 8 and 16 years. Exclusion criteria: medication with known psychotropic effects, diagnosis of psychiatric disorders (e.g., attention deficit disorder, anxiety, autism, or depression), visual impairment that was not corrigible with glasses, or history of traumatic brain injury treated by a physician. Written informed consent was obtained prior to participation. Some of the participants returned for retest; however, retest data is beyond the scope of this article.

### Procedure

All participants underwent three tests administered in a strict order, by trained examiners (M-NK, UJ-R, SR, MS, CH, and LC): (1) Test of Non-verbal Intelligence [TONI-4, a non-verbal, untimed matrix reasoning test validated and standardized in the United States (Brown et al., 2010)]; (2) standard (written version) SDMT; (3) c-SDMT with constant symbol-digit key. For both the SDMT and the c-SDMT, errors were recorded. The assessment was performed either in the patient’s room or, for the outpatient participants, in a seminar room at the Children’s University Hospital of Bern.

### Statistical Analyses

All analyses were done using IBM SPSS Statistics 25.0. To examine the effect of hospitalization on the performance of IPS and the interaction between “groups” (inpatient vs. outpatient) and “age groups” (8–11 years vs. 12–16 years), we used a multivariate analysis of covariance (MANCOVA). “IQ” was set as covariate, and “group” and “age group” as fixed factors. The dependent variables were “SDMT” (total score) and “c-SDMT” (total trial time).

A repeated measures analysis of variance (repeated measures ANOVA) was performed to examine within-trial variation in reaction time between the eight trials of the c-SDMT for inpatient and outpatient participants. In this analysis, the eight single trial times were set as within-subject variables and “group” as the between-subjects factor in order to compare the two groups. Contrasts were adjusted to polynomial in order to compare all the eight trials with one another.

All assumptions were met [independent random sampling, level of variables, multicollinearity (all correlations <0.90), normality (Shapiro–Wilk test *p* = 0.329), homogeneity of variances (Levene’s test *p* = 0.807)] for the SDMT, but normality and homogeneity of variances were violated for the c-SDMT. Since non-parametric alternatives are rare for MANCOVA and both MANCOVA and ANOVA are considered fairly robust procedures if cell sizes are >20, we chose to stick to parametric testing.

A *p*-value of <0.05 was considered statistically significant.

## Results

Demographic characteristics are shown in Table 1. No significant difference between the outpatient and the inpatient participants was detected. Since errors were few in both the SDMT (group mean 0.75) and the c-SDMT (group mean 1.2), they were not further attended in our analyses.

**TABLE 1:** Demographic data on the outpatient and inpatient participants.

	Outpatient *n* = 38 (44.2%)	Inpatient *n* = 48 (55.8%)	*t*-test or χ ^2^	*p*-value
Age in years, mean (*SE*)	11.50 (0.40)	12.10 (0.34)	*t* = -1.15	0.25
Gender, *n* (%)			χ^2^ = 0.24	0.63
Female	17 (44.7)	24 (50)		
Male	21 (55.3)	24 (50)		
Non-verbal IQ, mean (*SE*)	106.63 (1.61)	104.35 (1.62)	*t* = 0.98	0.33

*SE, standard error; *t*, *t*-test; χ^2^, chi-squared test.*

### Effect of Hospitalization on IPS Performance

A MANCOVA (Table 2) was used to examine the effect of hospitalization on IPS performance. Pillai’s Trace was used to interpret multivariate tests, because Box’s test of equality of covariance matrices was significant (*p* = 0.001). The combined dependent variables (IPS of the SDMT and the c-SDMT) were not significantly affected by the covariate “IQ” [*F*(2,80) = 0.09, *p* = 0.92] nor by the interaction effect (“age group” × “group”) [*F*(2,80) = 1.05, *p* = 0.35]. The main effects of “age group” [*F*(2,80) = 43.99, *p* < 0.001] and “group” [*F*(2,80) = 4.28, *p* = 0.02] on IPS were significant. This means that IQ scores did not correlate with IPS and that the effect of hospitalization did not differ between the age groups, whereas both age and hospitalization *per se* were associated with differences in IPS.

**TABLE 2:** MANCOVA results.

		*df*	*SS*	*F*	*p*	η *^2^*
SDMT	Main effects					
	Age group	1	5195.62	82.56	< 0.001	0.50
	Group	1	269.52	4.28	0.04	0.05
	Age group * group	1	112.63	1.79	0.19	0.02
	Covariates					
	IQ	1	3.38	0.05	0.82	0.001
c-SDMT	Main effects					
	Age group	1	38946.47	56.52	< 0.001	0.41
	Group	1	121.40	0.18	0.68	0.002
	Age group * group	1	1112.431	1.614	0.21	0.02
	Covariates					
	IQ	1	12.73	0.02	0.89	< 0.001

*df, degrees of freedom; SS, sum of squares; *F*, *F*-ratio; η^2^, (Partial) eta-squared.*

Univariate analyses were used to further examine the relationship between age group and group and the SDMT and c-SDMT, after adjusting for the non-significant effect of the covariate (IQ). The effect of age group was still significant in univariate tests for the SDMT [*F*(1,81) = 82.56, *p* < 0.001] and for the c-SDMT [*F*(1,81) = 56.52, *p* < 0.001]. By contrast, univariate effects of “group” were significant for the SDMT [*F*(1,81) = 4.28, *p* = 0.04], but not for the c-SDMT [*F*(1,81) = 0.18, *p* = 0.68]. This showed that outpatient and inpatient participants performed differently in the SDMT and, thus, hospitalization affects the results of this test. Table 3 illustrates that outpatients performed better than inpatients. In contrast, outpatient and inpatient participants did not differ significantly in their c-SDMT performance indicating that hospitalization does not affect the results of the c-SDMT.

**TABLE 3:** Descriptive statistics of c-SDMT and SDMT by group, adjusted for IQ.

	SDMT (total score) adjusted mean (standard error)	c-SDMT (total trial time) adjusted mean (standard error)
Inpatient group	37.153 (1.293)	130.064 (3.852)
Outpatient group	40.763 (1.293)	132.487 (4.287)
Age group 8–11 years	31.077 (1.254)	152.853 (4.151)
Age group 12–16 years	46.839 (1.198)	109.698 (3.966)

### Within-Trial Variation of the c-SDMT in Outpatient and Inpatient Populations

**FIGURE 1:**
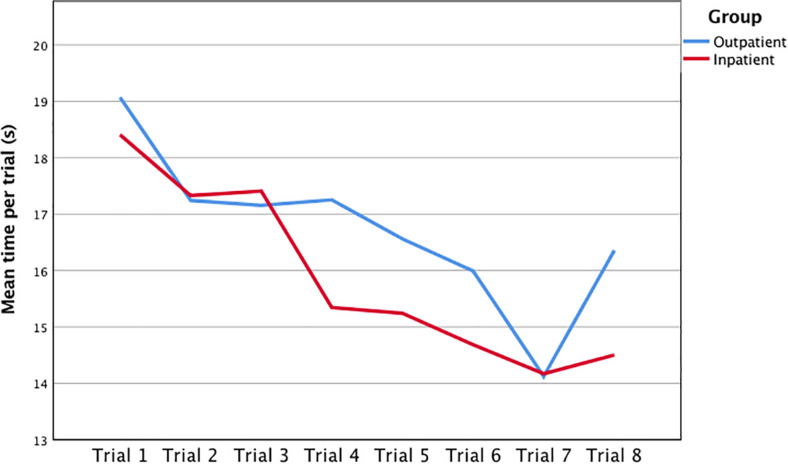
Mean time per trial (1–8) of the computerized symbol digit modalities test (c-SDMT) by group.

A one-way repeated measures ANOVA was conducted to compare the effect of “group” (outpatient vs. inpatient) on IPS performance from trial one to trial eight of the c-SDMT. Mauchly’s test indicated that the assumption of sphericity had been violated [χ^2^(27) = 107.38, *p* < 0.001] and degrees of freedom were therefore corrected using Greenhouse-Geisser estimates of sphericity (ε = 0.65). There was a significant group effect [Wilks’ lambda = 0.43, *F*(7,78) = 14.62, *p* < 0.001], showing that IPS performance in the c-SDMT across trials one to eight significantly differs between inpatient and outpatient groups. In tests of within-subject effects with the corrected Greenhouse-Geisser, c-SDMT performance over the eight trials was also significant [*F*(4,52) = 22.88, *p* < 0.001]. In general, the participants got faster from trial one to trial eight (Table 4 and Figure 1). However, the interaction of c-SDMT performance across trials and “group” did not reach statistical significance [*F*(4,52) = 2.19, *p* = 0.06]. In other words, the outpatient and inpatient groups did not significantly differ in their changes of performance speed from trial one to trial eight.

**TABLE 4:** Descriptive statistics trials 1–8 of the c-SDMT.

	Group	Mean	*SD*	*n*
c-SDMT T1	Outpatient	19.07	6.57	38
	Inpatient	18.41	6.13	48
	Total	18.70	6.30	86
c-SDMT T2	Outpatient	17.24	5.13	38
	Inpatient	17.33	5.15	48
	Total	17.29	5.11	86
c-SDMT T3	Outpatient	17.16	5.14	38
	Inpatient	17.41	4.93	48
	Total	17.30	4.99	86
c-SDMT T4	Outpatient	17.25	5.82	38
	Inpatient	15.34	3.56	48
	Total	16.19	4.76	86
c-SDMT T5	Outpatient	16.56	4.77	38
	Inpatient	15.24	3.64	48
	Total	15.82	4.20	86
c-SDMT T6	Outpatient	15.99	5.65	38
	Inpatient	14.69	4.10	48
	Total	15.26	4.86	86
c-SDMT T7	Outpatient	14.12	3.66	38
	Inpatient	14.17	3.54	48
	Total	14.15	3.57	86
c-SDMT T8	Outpatient	16.36	6.37	38
	Inpatient	14.50	4.03	48
	Total	15.32	5.25	86

*n, subsample size; SD, standard deviation.*

## Discussion

The SDMT and the c-SDMT are two screening tools for IPS. For the SDMT, associations with sex (Kiely et al., 2014), age (Arango-Lasprilla et al., 2017), psychological and health conditions (Kiely et al., 2014), as well as with education and IQ score (Vernon, 1983) have been reported. For the c-SDMT, only a correlation between age and IPS performance has been examined (Akbar et al., 2011; Bigi et al., 2017). The present study therefore evaluated whether hospitalization (H1a,b) and intelligence (H2) had an effect on performance of IPS in the c-SDMT versus the SDMT. The main findings of our study are that hospitalization had an impact on performance in the SDMT but not in the c-SDMT whereas IQ did not influence the results in either the SDMT or c-SDMT.

First and foremost, our results showed a significant effect of hospitalization on IPS performance as assessed in the written SDMT. Inpatient participants performed more slowly than outpatient ones. This could be interpreted as impairment of IPS performance by hospitalization through its environmental, psychological, and physical impacts (Crnkoviæ et al., 2009; Rokach, 2016). No such hospitalization effect was observed in the performance in the c-SDMT, confirming our hypothesis. Outpatient and inpatient participants performed similarly. To date, no data describing a possible effect of hospitalization on IPS are available to compare with our findings. The lack of an effect of hospitalization in the c-SDMT is probably due to the minimal motor requirements of this tool. Unlike the written SDMT, the c-SDMT does not require participants to write. Thus, we speculate that the hospitalization effect would be similarly minimized for the oral version of the SDMT. Another explanation, however, could be that less visual attention is needed in the computer-based test version, since only one trial is shown at a time.

Furthermore, the c-SDMT provides more detailed information about the within-test variation and therefore probably about attention, procedural learning and cognitive fatigability during the test (Bigi et al., 2017). Concerning the within-test variation of performance speed in the c-SDMT, no significant difference could be found between the inpatient and outpatient groups. Participants in both groups became faster from trial one to trial eight. Therefore, an impact of hospitalization by itself can be excluded, not only on the c-SDMT in general, but also on the within-test variation of performance speed. This confirms the findings of Bigi et al. (2017) who showed that the healthy control group could maintain attention and become faster between the trials. In contrast, the multiple sclerosis (MS) patients in their study showed an improvement in speed at the beginning, but could not keep this improvement up, and became slower during the last three trials (Bigi et al., 2017). This reveals another unique and useful advantage of the c-SDMT in a clinical setting over classical paper-and-pencil tests, as there is no within-test information to be acquired in the latter.

Secondly, our results indicated no association between SDMT or c-SDMT scores and the IQ score, i.e., faster IPS did not correlate with higher IQ scores. This is in contrast to the findings of Neubauer and Bucik (1996) and Rindermann and Neubauer (2004), who found a correlation between IPS and fluid intelligence (Neubauer and Bucik, 1996; Rindermann and Neubauer, 2004). But Fry and Hale (1996) found no significant direct effect of IPS on fluid intelligence. They described the association as more like a developmental cascade: IPS increases with age and correlates with better working memory. Furthermore, working memory correlates with fluid intelligence (Fry and Hale, 1996). These studies used different tools to measure fluid intelligence and IPS, which could have led to these conflicting results. One explanation for our findings could be that we used a fluid intelligence test (TONI-4) without a time limit, as the TONI-4 measures fluid intelligence independent of IPS (Brown et al., 2010). This might be why the IQ scores did not affect performance in either the SDMT or in the c-SDMT. In other studies using intelligence scales with a time component (such as Wechsler Scales), participants with faster IPS tended to have higher IQ scores (Vernon, 1983). In addition, both the SDMT and the c-SDMT only require a little cognitive effort. This is in line with the findings of Miller and Vernon (1992) who reported a smaller correlation between intelligence and reaction time once they had controlled for short-term memory (Miller and Vernon, 1992).

Additionally, our results revealed that age is the best predictor of IPS performance in the c-SDMT as well as in the SDMT. These findings are consistent with the results of previous studies (Hale, 1990; Fry and Hale, 2000; Bigi et al., 2017). Hale (1990) showed that for all the information processing tasks tested in her analyses (choice reaction time, letter matching, mental rotation, and abstract matching), that 12-year-old participants were faster than the younger group of 10-year-olds, but always slower than the 15-year-olds, who in turn did not differ from adults (Hale, 1990). Bigi et al. (2017) documented a similar age profile for the c-SDMT (Bigi et al., 2017).

Our results showed no significant difference in the effect of age between the outpatient and inpatient group. In other words, both outpatient and inpatient participants demonstrated improvement in IPS performance with increasing age.

The differences between the SDMT and the c-SDMT are crucial. In contrast to classical paper-and-pencil tests, the c-SDMT could more accurately reflect real impairment, because it is not influenced by the effect of hospitalization. The results of paper-and-pencil tests could be influenced by both motor impairment and hospitalization and could therefore be misleading. Therefore, in a clinical setting, the c-SDMT has benefits compared to classical paper-and-pencil tests of IPS: it is fairer for an inpatient population and no motor dexterity is needed making it suitable, for example, for paralyzed patients or patients with a broken arm. In addition, it provides a measure of trial-to-trial variation of IPS, which may be valuable information in some neurological conditions. Finally, all aspects are digitally recorded, the test can be administered anywhere, and is thus an easy-to-use bedside tool.

Our study is not without limitations. To reduce bias due to different settings, all participants were tested at the University Children’s Hospital Bern. Therefore, the sample size was relatively small, as the participants came from around the area of Bern and did not represent the whole country with its cultural and linguistic differences. In our current validation studies, we continue to sample healthy participants to pool our data for re-examination and confirmation of the issues discussed in this paper. Furthermore, unlike previous studies (Arango-Lasprilla et al., 2017), we did not take into account the educational level of the participants nor of their parents, as Switzerland has different educational systems in different parts of the country.

## Conclusion

Our findings suggest that performance in both SDMT and c-SDMT is independent of intelligence. The c-SDMT is a valid alternative to the classical paper-and-pencil tests of IPS, with the benefits of not requiring motor dexterity, not being influenced by hospitalization, and delivering more detailed information about within-test variation of IPS.

## Data Availability Statement

The raw data supporting the conclusions of this article will be made available by the authors, without undue reservation.

## Ethics Statement

The studies involving human participants were reviewed and approved by the Gesundheits- und Fürsorgedirektion des Kantons Bern, Kantonale Ethikkommission für die Forschung, Bern, Switzerland. Written informed consent to participate in this study was provided by the participants’ legal guardian/next of kin.

## Author Contributions

M-NK and UJ-R performed the acquisition of the data, analysis and interpretation of the data, and draft of the manuscript. SR, CH, MS, and LC were involved in the data acquisition. SB and KL drafted and supervised the study and critically revised the manuscript for intellectual content. All authors were involved in the conceptualization and design of the study, and read and approved the manuscript.

## Conflict of Interest

The authors declare that the research was conducted in the absence of any commercial or financial relationships that could be construed as a potential conflict of interest.

## Abbreviations

SDMT: symbol digit modalities test
c-SDMT: computer-based symbol digit modalities test
IPS: information processing speed
TBI: traumatic brain injury.
